# IL-21 Signaling in Immunity

**DOI:** 10.12688/f1000research.7634.1

**Published:** 2016-02-26

**Authors:** Warren J. Leonard, Chi-Keung Wan

**Affiliations:** 1 Laboratory of Molecular Immunology and the Immunology Center, National Heart, Lung, and Blood Institute, National Institutes of Health, Bethseda, Maryland, 20892, USA

**Keywords:** cytokine, IL-21, Immunosuppression, cancer immunotherapy, B-cell differentiation, T-cell differentiation

## Abstract

IL-21 is a type I cytokine produced by T cells and natural killer T cells that has pleiotropic actions on a wide range of immune and non-immune cell types. Since its discovery in 2000, extensive studies on the biological actions of IL-21 have been performed *in vitro* and *in vivo*. Recent reports describing patients with primary immunodeficiency caused by mutations of *IL21* or *IL21R* have further deepened our knowledge of the role of this cytokine in host defense. Elucidation of the molecular mechanisms that mediate IL-21’s actions has provided the rationale for targeting IL-21 and IL-21 downstream mediators for therapeutic purposes. The use of next-generation sequencing technology has provided further insights into the complexity of IL-21 signaling and has identified transcription factors and co-factors involved in mediating the actions of this cytokine. In this review, we discuss recent advances in the biology and signaling of IL-21 and how this knowledge can be potentially translated into clinical settings.

## Introduction

**Figure 1.**
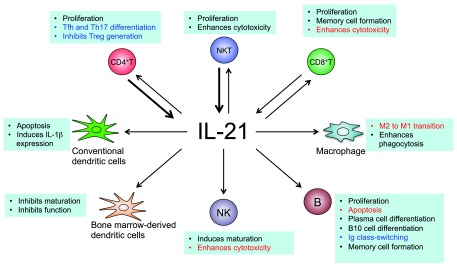
Sources of IL-21 and its major biological actions in different immune cell types. IL-21 is produced mainly by CD4 ^+^ T cells and NKT cells (see bold arrows), but it is also produced by CD8 ^+^ T cells. IL-21 acts on both lymphoid and myeloid populations and can positively or negatively regulate immune responses depending on the context. The text in red indicates biological actions that make IL-21 a potential anti-cancer agent: it enhances the cytotoxic actions of CD8 ^+^ T cells and NK cells, induces apoptosis of B cell lymphoma cells, and promotes the M2 to M1 transition of the tumor-associated macrophages. The text in blue indicates actions of IL-21 that may contribute to autoimmune diseases: differentiation of Tfh and Th17 cells, inhibition of Treg generation, and the production of auto-antibodies. Thus, blocking IL-21 signaling has promising therapeutic potential.

IL-21 is a pleiotropic type I cytokine that is produced mainly by T cells and natural killer T (NKT) cells. This cytokine has diverse effects on a broad range of cell types including, but not limited to, CD4 ^+^ and CD8 ^+^ T cells, B cells, macrophages, monocytes, and dendritic cells (DCs) ^1^ ( Figure 1). The functional receptor for IL-21 is composed of the IL-21 receptor (IL-21R) and the common cytokine receptor *γ* chain ( *γ*_c_), which is also a subunit of the receptors for IL-2, IL-4, IL-7, IL-9, and IL-15. Mutations of *γ*_c_ in humans result in X-linked severe combined immunodeficiency (XSCID), a disease characterized by the absence of T cells and natural killer (NK) cells, and with B cells that are normal in number but non-functional ^2^. It is now clear that defective IL-21 signaling substantially explains the defective B-cell function in this disease ^3, 4^. In the past few years, the use of next-generation sequencing technology, particularly chromatin immunoprecipitation coupled with DNA sequencing (ChIP-Seq) and RNA-Seq, has provided insights into the complexity of cell type-specific IL-21-mediated signaling and helped to identify the transcription factors and co-factors involved ^5^. The pathogenic role of IL-21 in various types of autoimmune diseases is supported by the use of animal models, clinical reports, and genome-wide association studies (GWAS) ^1^. Moreover, reports describing patients with primary immunodeficiency caused by *IL21* or *IL21R* mutations underscore the critical role of IL-21 in host defense *in vivo* in humans ^6– 9^. Knowledge of the biological functions of IL-21 has led to clinical trials using this cytokine alone or in combination with other agents in treating metastatic cancers, and blocking antibodies to IL-21R are now being evaluated in clinical trials for the treatment of autoimmune diseases. Interestingly, discovery of the immunosuppressive actions of IL-21 suggests that this cytokine is a “double-edged sword” – IL-21 stimulation may lead to either the induction or suppression of immune responses, so that both stimulatory and suppressive effects of IL-21 must be considered during the clinical use of IL-21-related immunotherapeutic agents. The biological effects of IL-21 are also influenced by the presence of other cytokines or signaling molecules in the microenvironment. Here, we review recent advances in our understanding of the biology and signaling of IL-21 and potential clinical applications.

## IL-21-activated STAT3 forms cell type-specific complexes for signaling

**Figure 2.**
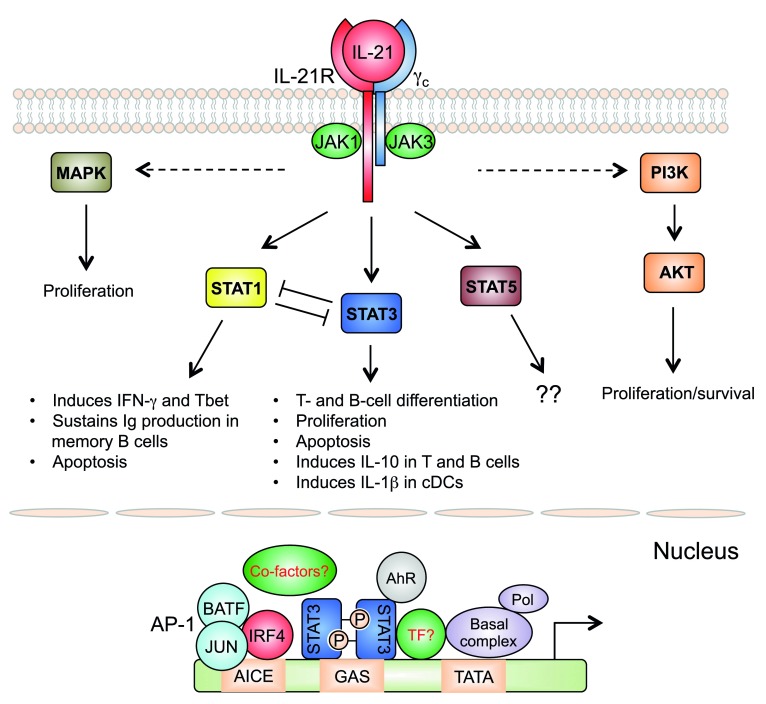
Signaling pathways activated by IL-21. IL-21 activates JAK-STAT, PI 3-kinase (PI3K), and MAP kinase (MAPK) pathways. STAT3 plays a major role in the biological actions of IL-21, but STAT1 also contributes to IL-21-regulated gene expression. Opposing actions of STAT1 and STAT3 are important for fine-tuning IL-21’s functions. The importance of IL-21-activated STAT5 is not known. MAPK and PI3K pathways contribute to the proliferative effect of IL-21. In T cells, after IL-21 stimulation, optimal STAT3-mediated gene regulation requires functional cooperation with IRF4, which binds together with AP-1 family proteins (predominately BATF and JUN family proteins), to regulate genes containing AP1-IRF4 composite elements (AICEs). AhR can also cooperate with STAT3 for gene regulation in T cells after IL-21 stimulation. Additional transcription factors (TFs) and co-factors may also be involved.

Analogous to other *γ*_c_ cytokines, IL-21 transduces molecular signals substantially via the Janus kinase and Signal Transducer and Activator of Transcription (JAK-STAT), phosphoinositide 3-kinase (PI3K), and mitogen-activated protein kinase (MAPK) pathways ^10^. IL-21 induces strong and sustained activation of STAT3, which is critical for its effects on B-cell and T-cell differentiation ^1^. The clinical significance of STAT3 in IL-21-mediated signaling has been confirmed in patients with *STAT3* mutations ^11– 15^. Studies have identified additional transcriptional factors and co-factors involved in IL-21-mediated signaling, with some of them forming complexes with STAT3 ^5^. In addition to STAT3, IL-21-induced T-helper (Th) 17 cell differentiation requires interferon regulatory 4 (IRF4), with *Irf4*-deficient CD4 ^+^ T cells having defects in IL-17 production after stimulation with IL-21 and TGF- *β* ^16^. ChIP-Seq analysis in both B cells and CD4 ^+^ T cells has revealed global cooperative activity of the IL-21-activated STAT3 with IRF4, with most regions with STAT3 binding activity also binding IRF4, and moreover IL-21-mediated, STAT3-dependent gene expression is diminished in the absence of IRF4 ^17^. IRF4 itself weakly binds to the DNA due to the presence of a carboxy-terminal auto-inhibitory domain, and in B cells, cooperative binding of IRF4 with ETS family proteins PU.1 or SPIB is known to increase the binding affinity of IRF4, resulting in the use of ETS-IRF4 composite elements, or EICEs. However, expression of PU.1 and SPIB is low in CD4 ^+^ T cells, leading to the unexpected discovery that, whereas B cells use EICEs, in T cells IRF4 cooperates with AP1 family proteins BATF and JUN and utilizes AP1-IRF4 composite elements (AICEs) ^18– 20^. Moreover, one study showed that cooperative activity of STAT3 and the aryl hydrocarbon receptor (AhR) is required for the expression of IL-22 in CD4 ^+^ T cells ^21^, indicating that the protein complexes activated by IL-21 likely involve additional proteins. These studies suggest that IL-21-mediated gene regulation often requires IRF4 in B and T cells, but IRF4 in T cells additionally complexes with AP-1 family proteins to regulate expression of certain genes, and perhaps this explains some T-cell-specific actions of IL-21 ( Figure 2).

A critical role for STAT3 in IL-21 signaling was also confirmed in patients with autosomal dominant hyper-IgE syndrome (AD-HIES), which is caused by loss-of-function mutations of STAT3. Consistent with a key role of IL-21 in Th17 cell differentiation, CD4 ^+^ T cells from these patients are not able to produce IL-17 *in vitro*, and, together with the defective IL-6 and IL-23R signaling ^22, 23^, this helps to explain their susceptibility to recurrent infections ^24^. Similarly, early studies showed that IL-21 together with CD40 engagement stimulates the differentiation of naïve B cells into IgG-producing plasma cells ^25^, and it was subsequently shown that naïve B cells from AD-HIES patients are not able to differentiate into IgG- or IgA-producing cells after IL-21 stimulation *in vitro* ^15^. However, the clinical manifestations in these patients cannot be explained solely by defective IL-21 signaling, as other cytokines including IL-6, IL-10, and IL-11 also strongly activate STAT3 for signal transduction ^26^.

IL-21 stimulation also leads to the activation of STAT1, at least in T cells, B cells, and conventional dendritic cells (cDCs), and recent studies have improved our understanding of the role of STAT1 in IL-21 signaling. IL-21-stimulated plasma cell generation remains intact in naïve B cells from STAT1 loss-of-function patients, but STAT1 plays a role in sustaining Ig production by differentiated memory B cells ^15^. In addition, IL-21 can enhance the cytotoxic activity of mouse CD8 ^+^ T cells by induction of T-bet, predominantly via STAT1 ^27^. Moreover, a recent study showed that ~10% of IL-21-regulated genes in pre-activated CD4 ^+^ T cells are dependent on STAT1, compared to ~40% being dependent on STAT3 ^28^. Strikingly, expression of some genes including Th1 signature genes *Ifng* and *Tbx21* by IL-21 are differentially regulated by STAT1 and STAT3, and IL-21-induced expression of *IFNG* and *TBX21* is enhanced in CD4 ^+^ T cells from AD-HIES patients and also modestly increased in CD4 ^+^ T cells from STAT1 gain-of-function patients ^28^. These findings suggest that an interplay between STAT1 and STAT3 may fine-tune IL-21-induced biological actions. This conclusion is also supported by the fact the STAT3 loss-of-function (AD-HIES) and STAT1 gain-of function patients share immunological phenotypes (e.g., defective IL-17 production in CD4 ^+^ T cells and impaired production of antigen-specific antibodies) and clinical manifestations (e.g., mucocutaneous candidiasis) ^12, 24, 29, 30^.

## Biological functions of IL-21 revealed from studies of patients with *IL21R* or *IL21* mutations

Patients with primary immunodeficiency caused by *IL21R* or *IL21* mutations have been described ^6– 9^, and their phenotypes have provided invaluable insights into the role of IL-21 in host defense. Patients with defective IL-21 signaling suffer from recurrent pulmonary infections, and patients with *IL21R* mutations, but not the single described patient with *IL21* mutations, additionally have cryptosporidiosis, leading to secondary cholangitis and liver disease. Infections with opportunistic pathogens may be due to the defects in both innate and adaptive immunity in these patients, as plasma cell and memory B-cell generation, as well as immunoglobulin class switching are impaired ^15^, while CD8 ^+^ T-cell proliferation and NK -cell cytotoxicity are also diminished. Interestingly, the patient with the *IL21* mutation did not have cryptosporidiosis but had early-onset inflammatory bowel disease (IBD) ^7^.

## Immunosuppressive effects of IL-21

The early onset IBD observed in the one *IL21*-mutated patient, alongside chronic diarrhea in some *IL21R*-deficient patients, was interesting, as multiple studies using animal models showed that IL-21 promotes the pathogenesis of IBD ^31^. However, IL-21 can also be immunosuppressive because of its ability to induce IL-10. IL-10 signaling is known to critically regulate immune homeostasis in the gut, and patients with *IL10* ^32^ or *IL10R* ^33^ mutations also develop severe early onset IBD. Interestingly, under Th1 priming conditions, the addition of IL-21 was shown to potently inhibit antigen-induced IL-2R *α* expression and cell cycle progression of naïve CD8 ^+^ T cells in a STAT3-mediated, IL-10-dependent fashion ^34^. Moreover, IL-27-mediated differentiation of IL-10-producing regulatory type 1 (Tr1) cells requires IL-21, c-Maf, and ICOS, with IL-21 acting as an autocrine factor to maintain Tr1 cells ^35^. A more recent report showed that IL-21 drives human cord blood T cells into IL-10-producing Th1 cells ^36^, suggesting that IL-21 can also exhibit immunosuppressive effects in humans. IL-21 together with CD40L induces human B cells to produce IL-10, particularly in memory B cells that have undergone immunoglobulin class switching ^37^, showing that the induction of IL-10 by IL-21 is not restricted to T cells. In fact, IL-10-producing regulatory B cells (B10 cells) can be greatly expanded *in vitro* by engagement of CD40 and IL-21 receptors, and transferring these cells into mice significantly inhibits disease symptoms in experimental autoimmune encephalomyelitis (EAE), a model of human multiple sclerosis ^38^. IL-21-derived B10 cells also express granzyme B, which degrades the T-cell receptor ζ-chain and limits T-cell proliferation ^39^, providing an additional mechanism by which these cells can suppress immune responses.

Furthermore, IL-21 can potently induce apoptosis of B cells ^25, 40, 41^ and cDCs ^42^, which may provide alternative mechanisms for its suppressive effect. Stimulation of mouse naïve B cells *in vitro* with IL-21 without co-stimulatory signals induces apoptosis via the induction of pro-apoptotic BIM expression ^40^ and suppression of pro-survival BCL2 and BCLXL ^41^. However, pre-activation of B cells with anti-CD40 and anti-IgM inhibits the apoptotic effect of IL-21 ^25, 40, 41^, and CD40 engagement combined with IL-21 drives B-cell differentiation to plasma cells via induction of BLIMP1 and stimulates Ig class-switching via STAT3 activation ^12, 25, 43, 44^. These results suggest that, analogous to IL-2-mediated activation-induced cell death in T cells ^45^, IL-21 might help to eliminate B cells that are activated in an antigenic non-specific fashion without the cognate antigen-specific or co-stimulatory signals.

IL-21 is known to inhibit the maturation and function of bone marrow-derived dendritic cells (BMDCs) ^46, 47^. It also inhibits the LPS-stimulated expression of pro-inflammatory cytokines IL-6 and IL-1 *β* by these cells ^46^. IL-21 can potently induce the apoptosis of cDCs via STAT3-mediated BIM induction ^42^. The apoptosis induced by IL-21 can be prevented by GM-CSF, which activates STAT5. Interestingly, ChIP-Seq analysis shows that STAT3 and STAT5 compete for DNA binding at the *Bim* locus, suggesting a direct competitive effect for these STAT proteins. Further investigation of the effect of IL-21 on cDCs revealed an unexpected role of IL-21 in IL-1 *β* expression via a NF- *κ*B-independent, STAT3-dependent pathway, with direct STAT3 binding identified in the *Il1b* locus in cDCs after IL-21 stimulation ^48^. These studies suggest that IL-21 has dual roles in DCs where it suppresses immune responses by inhibiting the maturation and actions of BMDCs and inducing apoptosis in cDCs, but promotes immune responses by inducing IL-1 *β* in cDCs.

There are extensive data indicating that IL-21 signaling promotes the pathogenesis of autoimmune diseases ^1^, including in animal models of type 1 diabetes ^49, 50^, systemic lupus erythematosus (SLE) ^51^, and experimental autoimmune uveitis ^52^. Moreover, the number of IL-21-producing CD4 ^+^ T cells is higher in patients with active SLE ^53^ and chronic rheumatoid arthritis (RA) ^54^, suggesting that blocking IL-21 signaling might serve to ameliorate these diseases. However, the effects of IL-21 can be complex, and IL-21 signaling in certain cell types can have protective effects as well. For example, in SLE-prone BXSB- *Yaa* mice, although selective ablation of IL-21R expression in B cells protects the mice from developing disease manifestations, IL-21 signaling supports the expansion of CD8 ^+^ suppressor T cells in these mice and, as a result, selective ablation of IL-21R in CD8 ^+^ T cells also promotes pathogenesis of the disease ^55^. Also, IL-21-induced IL-22 expression in CD4 ^+^ T cells may play a protective role in the DSS-induced colitis model ^21^. Thus, although blocking IL-21 signaling is currently under evaluation in early clinical trials for the treatment of autoimmune disease, it conceivably could have mixed effects depending on the context in individual patients.

## IL-21 is a promising immunotherapeutic agent for cancer

Activation of the cytotoxic programs in NK cells and CD8 ^+^ T cells is key for cancer immunotherapy, and consequently early studies provided compelling evidence that IL-21 is a promising immunotherapeutic agent for this disease ^56^. IL-21 promotes maturation, enhances cytotoxicity, and induces production of IFN- *γ* and perforin by NK cells ^57, 58^. Correspondingly, cytolytic activity induced by IL-21 significantly inhibits the growth of B16 melanoma ^58, 59^. Moreover, IL-21 together with IL-15 expands antigen-specific CD8 ^+^ T-cell numbers and their effector function, resulting in tumor regression ^60^. In addition, cancer cells over-expressing IL-21 cannot graft to the host and are rapidly eliminated ^61– 64^, and local delivery of IL-21 into the tumor microenvironment in a breast tumor model was shown to switch tumor-associated macrophages from the M2 phenotype to a tumor-inhibiting M1 phenotype, which rapidly stimulates T cell responses ^65^. These studies suggest that IL-21 can “rejuvenate” multiple effector cells in the tumor microenvironment and thus that this cytokine might be used alone or in combination with other therapeutic agents in a clinical setting. Indeed, clinical trials are underway, with encouraging results ^1^. In one phase II study in which IL-21 was used as a single agent to treat patients with metastatic melanoma who had not received prior systemic therapy, a response rate of 22.5% was achieved ^66^. Another phase 1/2 study investigated the effects of IL-21 combined with the tyrosine kinase inhibitor sorafenib for treating metastatic renal cell carcinoma, and a disease control rate of 82% was achieved ^67^.

IL-21 is known to directly induce apoptosis in certain types of lymphoma. *In vitro* studies showed that IL-21 potently induces apoptosis of diffuse large B-cell lymphoma ^68^, mantle cell lymphoma ^69, 70^, and chronic lymphocytic leukemia ^71^ cells via activation of STAT3 or STAT1, leading to the altered expression of BCL2 family proteins and the activation of caspases. Besides its direct apoptotic effect, IL-21 alone or combined with anti-CD20 monoclonal antibody (mAb) (rituximab) can also indirectly kill the IL-21-insensitive cancer cells by activating NK cell-dependent cytotoxic effects ^69, 72^. Based on these results, a phase I study combined IL-21 with rituximab for treating 19 patients with indolent B-cell malignancies, and 42% of patients obtained complete or partial responses ^73^. Unlike IL-2, injection of high-dose IL-21 does not cause capillary leak syndrome *in vivo* ^74^ and was well tolerated.

Adoptive transfer of *in vitro* expanded tumor antigen-specific CD8 ^+^ T cells into patients is another promising anti-cancer strategy. When leukemia antigen-specific CD8 ^+^ T cells purified from an HLA-matched donor were cultured with IL-21 *in vitro* and then infused into a patient, the CD8 ^+^ T cells showed a long-lived memory phenotype compared to the cells not treated with IL-21. Patients receiving the IL-21-cultured cells had a marked decrease in leukemic cells and a sustained complete remission ^75^. These results indicate that IL-21 may be a potent adjuvant for cell-based cancer immunotherapy.

## Critical role of IL-21 in chronic viral infection

The vital role of IL-21 in anti-viral immunity has been demonstrated mainly in studies using models of chronic lymphocytic choriomeningitis virus (LCMV) infection ^76– 79^. During chronic LCMV infection, IL-21 is produced by CD4 ^+^ T cells, which sustains CD8 ^+^ T cell expansion and production of IFN- *γ*, TNF- *α*, and IL-2 ^78^. Correspondingly, mice lacking IL-21 or IL-21R show diminished CD8 ^+^ T cell clonal expansion, increased exhaustion, and persistent high serum viral titers ^76– 78^, indicating that IL-21 directly acts on CD8 ^+^ T cells to limit chronic viral infections. In addition, IL-21 can also indirectly activate the anti-viral activity of CD8 ^+^ T cells by suppressing the expansion of Treg cells during chronic LCMV infection ^79^. Although the requirements for IL-21 signaling in host defense during acute viral infection are less stringent, studies using the Armstrong (acute) strain of LCMV or vaccinia virus showed that IL-21 signaling is essential for the survival of activated CD8 ^+^ T cells and generation of long-lived memory cells ^80, 81^. Moreover, IL-21 acting on B cells and CD4 ^+^ T cells is critical for generating long-lived plasma cells after infection with an acute strain of LCMV, vesicular stomatitis virus, and influenza virus, highlighting the importance of IL-21 in humoral immunity during viral infection ^82^.

## IL-21 as a potential vaccine adjuvant

The biological actions of IL-21 on NK cells, CD8 ^+^ T cells, and B cells described above, as well as its potent anti-viral property shown in mouse models, make it an attractive candidate for use as a vaccine adjuvant. Indeed, IL-21 has been shown to play important roles in controlling disease progression after human immunodeficiency virus (HIV) infection. Serum levels of IL-21 are significantly reduced in HIV-infected patients and correlate with CD4 ^+^ T-cell counts ^83^. Among different disease-status groups of HIV-infected patients, only the elite controllers maintain normal production of IL-21, and IL-21-producing CD4 ^+^ T cells are decreased in HIV-infected viremic patients or patients with progressive disease ^84, 85^. Interestingly, CD8 ^+^ T cells in HIV-infected patients produce IL-21, and the frequencies of these cells are closely associated with viral control ^86, 87^, suggesting that the loss of IL-21 production correlates with disease progression. Correspondingly, recent studies showed that T follicular helper (Tfh) cells, which are the major source of IL-21, are the most efficiently infected by HIV among different CD4 ^+^ T-cell subtypes ^88^, and defective Tfh function results in impaired humoral immunity against HIV ^89^. The potential use of IL-21 therefore has been investigated using non-human primate models. Similar to the *ex vivo* effects of IL-21 on NK and CD8 ^+^ T cells isolated from HIV-infected patients ^90, 91^, injecting IL-21 into simian immunodeficiency virus (SIV)-infected rhesus macaques increases cytotoxic activity and the production of granzyme B and perforin by these cells ^92, 93^. The frequencies of SIV-specific CD8 ^+^ T cells, peripheral blood CD27 ^+^ memory B cells, and serum SIV antibodies are also increased after IL-21 administration ^92^. Intriguingly, IL-21 injection alone or in combination with anti-retroviral therapy in SIV-infected rhesus macaques leads to the restoration of intestinal Th17 cells, which is associated with reduced microbial translocation from the intestinal lumen into the systemic circulation, systemic inflammation, and morbidity ^93– 95^. Together, these studies indicate that IL-21 can be used as an adjuvant for anti-viral therapies.

## Concluding remarks

IL-21 is being intensely studied, with new information emerging on its biological effects, its signaling mechanism(s), and clinical potential. Studies in patients with mutations in *IL21*, *IL21R* and *STAT3* confirmed the major roles of IL-21-activated STAT3 signaling in T-cell and B-cell differentiation and also revealed roles for its STAT3-independent signaling. ChIP-Seq analysis has successfully identified protein complexes activated by IL-21, which help to explain the cell-type-specific effects of this cytokine. Because multiple cytokines including IL-6 and IL-10 also activate STAT3, it will be interesting to know whether these cytokines activate formation of the same complexes as IL-21 or whether there are cytokine-specific complex(es). As discussed above, IL-21 is a promising agent for treating cancers. Clinical trials using IL-21 as an adjuvant for cell-based cancer immunotherapy have been encouraging. In addition, clinical trials using blocking IL-21R mAb for autoimmune diseases are ongoing ^1^. Moreover, *in vitro* expansion of regulatory B10 cells by IL-21 is potent in a mouse model and thus may have potential for human autoimmunity as well, an area for future research. In mouse models, IL-21 has been shown to play critical role(s) in the development of graft- *versus*-host disease (GVHD) ^96– 100^, and future clinical investigation of the possibility of using IL-21-blocking agents to treat GVHD is needed. Overall, the study of the biological actions and signaling mechanisms of IL-21 has provided critical basic insights and the rationale for clinical evaluation of IL-21, both in cancer and in the use of antibodies to IL-21 in autoimmunity and potentially other diseases as well.
